# The Relative Impacts of Disease on Health Status and Capability Wellbeing: A Multi-Country Study

**DOI:** 10.1371/journal.pone.0143590

**Published:** 2015-12-02

**Authors:** Paul Mark Mitchell, Hareth Al-Janabi, Jeff Richardson, Angelo Iezzi, Joanna Coast

**Affiliations:** 1 Health Economics Unit, Institute of Applied Health Research, University of Birmingham, Birmingham, United Kingdom; 2 Centre for Health Economics, Monash University, Melbourne, Australia; 3 School of Social and Community Medicine, University of Bristol, Bristol, United Kingdom; Weill Cornell Medical College in Qatar, QATAR

## Abstract

**Background:**

Evaluations of the impact of interventions for resource allocation purposes commonly focus on health status. There is, however, also concern about broader impacts on wellbeing and, increasingly, on a person's capability. This study aims to compare the impact on health status and capability of seven major health conditions, and highlight differences in treatment priorities when outcomes are measured by capability as opposed to health status.

**Methods:**

The study was a cross-sectional four country survey (n = 6650) of eight population groups: seven disease groups with: arthritis, asthma, cancer, depression, diabetes, hearing loss, and heart disease and one health population ‘comparator’ group. Two simple self-complete questionnaires were used to measure health status (EQ-5D-5L) and capability (ICECAP-A). Individuals were classified by illness severity using condition-specific questionnaires. Effect sizes were used to estimate: (i) the difference in health status and capability for those with conditions, relative to a healthy population; and (ii) the impact of the severity of the condition on health status and capability within each disease group.

**Findings:**

5248 individuals were included in the analysis. Individuals with depression have the greatest mean reduction in both health (effect size, 1.26) and capability (1.22) compared to the healthy population. The effect sizes for capability for depression are much greater than for all other conditions, which is not the case for health. For example, the arthritis group effect size for health (1.24) is also high and similar to that of depression, whereas for the same arthritis group, the effect size for capability is much lower than that for depression (0.55). In terms of severity within disease groups, individuals categorised as 'mild' have similar capability levels to the healthy population (effect sizes <0.2, excluding depression) but lower health status than the healthy population (≥0.4).

**Conclusion:**

Significant differences exist in the relative effect sizes across diseases when measured by health status and capability. In terms of treating morbidity, a shift in focus from health gain to capability gain would increase funding priorities for patients with depression specifically and severe illnesses more generally.

## Introduction

All health care systems in all societies are constrained by the availability of resources. All have to set priorities and, whilst there are numerous suggestions about how this should be done [1], importance is generally attached to obtaining good outcomes from interventions, often alongside a comparison of cost in an economic evaluation. The question of what constitutes a good outcome is fundamental. Commonly it is equated with improvements in health status. In health economics this is measured through instruments weighted using population preferences that are known as health related utility. Health related utility allows for the comparison of diverse health states on a cardinal (interval or ratio) scale anchored at utility = 1 (best possible health) and utility = 0 (death) representing people’s preferences for different states of health-related quality of life [2].

There are, however, arguments for exploring measures of outcome including subjective wellbeing [3] and capability [4–8]. Broader outcome measures facilitate evaluation across programme areas, such as social care, public health, crime and education as well as health care. Measures of subjective wellbeing essentially focus on happiness but an exclusive focus upon this can be criticised as too limiting, as hedonic adaptation results in the disregard of serious limitations to a person’s abilities [9]. The focus of the capability approach is on “not just what a person actually ends up doing, but also on what she is in fact able to do, whether or not she chooses to make use of that opportunity” [10]. There has been a notable upsurge in interest in applying the approach to resource allocation decisions in health care [5,11–17], as well as increasing numbers of empirical studies employing the approach [18–21].

A change in focus—from health gain to capability gain—would potentially raise the priority of those activities that most affect the capability and, conversely, reducing the priority of activities which have little or no effect upon capability. This would therefore lead to a reallocation of healthcare resources. The objective of the present study is to compare the effect on health status and capability of major health conditions and therefore to determine whether a shift in evaluative focus from health status to capability is likely to have a major impact upon resource allocation priorities.

## Methods

### Study Design and Participants

The study uses data from a Multi Instrument Comparison (MIC) cross sectional survey of individuals in eight health categories (http://www.aqol.com.au). The survey was conducted in six countries of which the four English speaking nations were chosen for the present analyses (as the choice of these countries did not raise problems associated with translation). Details of the two countries (Germany and Norway) excluded from this study can be found elsewhere [22]. As well as a healthy population, seven broad disease groups were targeted, viz, persons with arthritis, asthma, cancer, diabetes, depression, hearing loss, and heart disease. These disease areas were chosen to represent major chronic conditions that result in significant burden of disease in developed countries [23,24].

### Instruments

Condition-specific instruments were used to measure the severity of each condition. Two instruments were selected to measure health status and capability. To this end, the measures were selected as (i) being short, simple measures often applied in clinical trials and regulatory decision making, and (ii) because both allow a meaningful index to be attributed to the level of health status/capability that is measured.

#### Health: EQ-5D-5L

The EQ-5D-5L is an updated version of the original EQ-5D-3L generic measure of health status [25,26]. The instrument is recognised as one of the most widely used generic measures of health status to generate quality-adjusted life-years (QALYs) [27]. It has been translated into 169 different languages. EQ-5D data are routinely collected in some countries as well as being used to inform healthcare decision-making [28]. The instrument has five dimensions (mobility, self-care, usual activities, pain/discomfort and anxiety/depression). Each dimension has five response levels (http://www.euroqol.org) [29]. Once a response level for each dimension is selected by the patient, general population values can then be attached to the patient’s current health state. These general population values are derived from previous time trade-off exercises with members of the UK general population to elicit the strength of preference for different health states over others [30,31]. The use of population values (which represent the average value of a sample of the general public), as opposed to patient values (which represent the value placed on a state by the individual in that state), is the preferred approach by health guidance bodies such as the National Institute for Health and Care Excellence (NICE) [32]. Population values are considered by some as a more appropriate method because it might allow for greater objectivity across disease areas and it incorporates the valuation of health benefits by citizens/taxpayers [33]. The general population approach to valuing health states means that all possible health states across a health service can be, in theory, compared to one another. Preliminary general population values for the EQ-5D-5L have been developed from the three level versions [31], as research is currently ongoing to derive new value sets for the five level version. The measure is anchored at 1 (best health) and 0 (death), with a minimum value of -0.594 for the UK value set. Values thus range from -0.594 to 1, with negative values representing states considered worse than being dead. There has been one major study to date assessing the validity of EQ-5D-5L across a variety of conditions [34].

#### Capability: ICECAP-A

The ICECAP-A instrument was designed to provide a summary measure of an individual’s capability wellbeing, for use in evaluating the benefits of health and social care interventions [35]. The ICECAP-A draws on the capability approach in terms of covering broad determinants of wellbeing through five questions about a person’s capability (as opposed to their functioning). There is discussion in the philosophical literature around the capability approach about how best to derive lists of capabilities for use in evaluation. Whilst some, such as Martha Nussbaum, prefer the notion of a set of capabilities that is fixed across all contexts [4], others, including Sen himself, see capabilities as more appropriately derived for particular contexts [10]. The descriptive system for the ICECAP-A instrument was developed in the UK using qualitative methods [35]. It contains five attributes of “*capability wellbeing*” (stability, attachment, autonomy, achievement and enjoyment), each with four levels (http://www.birmingham.ac.uk/icecap). The attributes aim to capture the capabilities that people value as distinct from the factors that determine capability (e.g. income, health) [35]. In doing so, the ICECAP-A measure aims to provide a broader conceptualisation of an individual’s wellbeing than solely relying on their health status. This approach to conceptualising capability states differ from those who argue for valuing health states through their impact on capability [15,36].

ICECAP-A UK population values have been developed [37]. The values for ICECAP-A rely on an approach known as best-worst scaling, that reduces the reliance on preferences used for measures like the EQ-5D that is challenged by the capability approach [38]. The measure is anchored at 1 (full capability) and 0 (no capability). Values can range from 0 to 1. The measure has been validated for the general adult UK population [39].

#### Condition-Specific Questionnaires

Table 1 summarises the condition-specific questionnaires used for the seven disease groups [40–47]. Global scores for each condition were required to judge the impact of increased disease severity on individual outcomes (see ‘Data Analysis’). For asthma, diabetes, depression and heart disease, the global score was obtained through a simple summation of all items on the questionnaire. For arthritis and cancer the global score was obtained through summing the subscales. For hearing loss a slightly more complex calculation was required to account for the particular format of the measure which records hearing loss both with and without the use of hearing aids. Specific methods for calculating the global scores for each condition-specific questionnaire are contained in the S1 Appendix. The overall method was tested for validity in relation to clinical severity levels for the depression questionnaires (the only questionnaires for which clinical cut-offs were available). Results of this validity test are presented in the S2 Appendix.

**Table 1:** Condition-specific questionnaires used for disease groups.

Arthritis	Asthma	Cancer	Depression^a^	Diabetes	Hearing Problems	Heart Disease
***AIMS2‒SF*** [40]	***AQLQ–Sydney*** [41]	***EORTC QLQ‒C30*** [42]	***DASS21*** [43]	***Diabetes‒39*** [45]	***APHAB*** [46]	***MacNew*** [47]
26 items	20 items	30 items	21 items	39 items	48 items^b^	27 items
5 sub-scales	4 sub-scales	7 sub-scales	3 sub-scales	5 sub-scales	4 sub-scales;	3 sub-scales
1. Physical	1. Breathlessness	1. Physical functioning	1. Stress	1. Energy and mobility	1. Ease of communication	1. Emotional
2. Symptom	2. Mood disturbances	2. Role functioning	2. Depression	2. Diabetes control	2. Reverberation	2. Physical
3. Affect	3. Social disruption	3. Emotional functioning	3. Anxiety	3. Anxiety and worry	3. Background noise	3. Social
4. Social interaction	4. Concerns for health	4. Cognitive functioning		4. Social burden	4. Aversiveness	
5. Work		5. Social functioning		5. Sexual functioning		
		6. Pain				
		7. Fatigue				
			***K10*** [44]			
			10 items			
			0 sub-scales			

AIMS2‒SF, Arthritis Impact Measurement Scales 2‒Short Form; APHAB, Abbreviated Profile of Hearing Aid Benefit; AQLQ-Sydney, Asthma Quality of Life Questionnaire; DASS21, Depression, Anxiety and Stress Scale 21 item; EORTC QLQ‒C30, European Organisation for Research and Treatment of Cancer Quality of Life Questionnaire‒Core 30; K10, Kessler Psychological Distress Scale 10 item; MacNew heart disease health related quality of life questionnaire;

^a^ Depression global scores reported using the DASS21.

^b^ APHAB consists of two sections, with hearing aid and without hearing aid consisting of 24 items each

### Data collection

The survey was conducted online with panel members using a global survey company, CINT Pty Ltd. The personal and medical details recorded by the company were used to recruit individuals from the disease groups and from the ‘healthy’ public, i.e. those who did not report any chronic disease and who obtained a score of at least 70 on a 100 point visual analogue scale measuring overall health. Quota sampling was conducted to obtain a ‘healthy’ public sample with age, sex and educational levels that was broadly representative of the general population. The only quota for disease groups was the total number sought in each disease group irrespective of age, sex and education. The survey sought a sample of 150 individuals per country in each disease area and a sample of 300 ‘healthy’ public per country to ensure statistical power for the initial study [22]. Individuals were asked to complete a relevant disease specific questionnaire to confirm the existence of the illness and to measure its severity.

The survey was conducted between February and May 2012. Ethics approval was obtained from Monash University Human Research Ethics Committee (MUHREC Approval CF11/1758: 2011 00074). At the start of the survey, a Participant Information and Consent form was provided. Proceeding with the survey was deemed as consent.

### Data Analysis

‘Edit criteria’, based on a comparison of duplicated or similar questions, were used to determine reliability and validity of the data. Exclusions were based upon several criteria. These included the existence of multiple IDs (people accessing the system and responding on more than one occasion); completion of the entire survey in less than 20 minutes—a time which made it virtually impossible for considered responses; inconsistent responses across a number of similar or identical questions; and misclassification of the healthy group (indicating very poor health or the presence of one of the indicated disease areas). Further detail on data collection and editing prior to the analysis undertaken in this study is provided elsewhere [22].

Statistical analysis was conducted with two main foci; to judge, first, the likely benefit of preventing conditions, and second, the likely benefit of treating (reducing the severity) of conditions. First, impacts that would be associated with potential prevention (i.e. avoided losses in health and capability from preventing onset of disease), were assessed by estimating the differences in health status and capability for those with conditions, relative to a healthy population. Second, impacts that might be associated with potential treatment (i.e. improvements in health and capability from, for example, a movement from moderate to mild disease severity), were assessed by estimating the differences in health status and capability for different severities of each conditions. The statistical packages used to analyse the data were Microsoft Excel 2010 and STATA 12.

#### Potential prevention impact comparing health and capability between disease and healthy population groups

Descriptive statistics were calculated for both outcomes for the seven disease groups and the healthy population. Values for capability and health responses were calculated using UK values.

To measure the differences between the healthy population and each disease group, Cohen’s d effect size was calculated (see Eq 1). This is the difference in means divided by the standard deviation of the two populations (e.g. A and B) under consideration [48] and is a useful method for comparing the same populations across two or more different instruments, even when the scales of the instruments differ [49]. A small effect size is generally considered to be at least 0.2, a medium effect size at least 0.5 and a large effect size 0.8 and above [48].

$$\frac{\left(mean\;A-mean\;B\right)}{Standard\;Deviation\left(A+B\right)}$$ (1)

#### Potential treatment impact comparing health and capability of different severities of condition

Individuals in disease groups were then categorised into condition severity levels by their responses to the condition-specific questionnaires. Not all condition-specific questionnaires have clinical cut-offs for severity levels; therefore a method for standardising the severity of different conditions and their relative impacts on health status and capability was required. Global scores for each of the condition-specific questionnaires were calculated and individuals categorised as having severe (global score below 0.4), moderate (global score between 0.4 and 0.7 inclusive) or mild (global score greater than 0.7) conditions according to this global score. This method has previously been used for the Arthritis Impact Measurement Scale 2—Short Form (AIMS2-SF) to compare across studies [50].

## Results

6,650 participants were initially included in the survey across the four countries, of whom 1,054 (15.8%) were excluded during the data cleaning exercise. Reasons for exclusion were inconsistent responses (484; 45.0%), completion in less than 20 minutes (440; 41.0%), misclassification (92; 8.6%) and multiple IDs (58; 5.4%). Additional exclusions were made for this analysis: 89 respondents from Australia were classified in further disease categories of COPD and stroke, not collected in other settings; 247 individuals were classified as healthy but had other conditions outside of those specified; and 12 did not complete the hearing-loss condition-specific questionnaire in sufficient depth to be classified for the purpose of this analysis. In total, 5,248 participants were included across Australia, Canada, UK and USA. Table 2 provides basic socio-demographic information for the different groups included in the analysis (see S3 Appendix for further country breakdown).

**Table 2:** Socio-demographic information for included participants (n = 5248).

	Sample Size	Sex	Age Groups						Country				Highest Education Attained		
	total	females	18–25	25–34	35–44	45–54	55–64	65+	Aus	Can	UK	US	High School	Diploma, Certificate or Trade	University
Healthy	965	513	122	189	189	181	139	145	200	269	234	262	359	310	296
	(18%)	(53%)	(13%)	(20%)	(20%)	(19%)	(14%)	(15%)	(21%)	(28%)	(24%)	(27%)	(37%)	(32%)	(31%)
Arthritis	640	440	9	38	58	146	227	162	163	139	159	179	230	248	162
	(12%)	(69%)	(1%)	(6%)	(9%)	(23%)	(35%)	(25%)	(25%)	(22%)	(25%)	(28%)	(36%)	(39%)	(25%)
Asthma	579	399	80	137	136	97	87	42	141	138	150	150	179	188	212
	(11%)	(69%)	(14%)	(24%)	(23%)	(17%)	(15%)	(7%)	(24%)	(24%)	(26%)	(26%)	(31%)	(32%)	(37%)
Cancer	577	349	7	23	47	106	217	177	154	138	137	148	197	195	185
	(11%)	(60%)	(1%)	(4%)	(8%)	(18%)	(38%)	(31%)	(27%)	(24%)	(24%)	(26%)	(34%)	(34%)	(32%)
Depression	617	416	64	158	142	148	85	20	146	145	158	168	215	214	188
	(12%)	(67%)	(10%)	(26%)	(23%)	(24%)	(14%)	(3%)	(24%)	(24%)	(26%)	(27%)	(35%)	(35%)	(30%)
Diabetes	641	297	9	46	64	153	226	143	168	144	161	168	221	245	175
	(12%)	(46%)	(1%)	(7%)	(10%)	(25%)	(37%)	(23%)	(27%)	(22%)	(25%)	(26%)	(34%)	(38%)	(27%)
Hearing	589	275	22	51	62	103	158	193	157	146	126	160	194	200	195
Loss	(11%)	(47%)	(4%)	(9%)	(11%)	(17%)	(27%)	(33%)	(27%)	(25%)	(21%)	(27%)	(33%)	(34%)	(33%)
Heart	640	259	24	33	40	109	216	218	149	154	167	170	228	247	165
Disease	(12%)	(40%)	(4%)	(5%)	(6%)	(17%)	(34%)	(34%)	(23%)	(24%)	(26%)	(27%)	(36%)	(39%)	(26%)
Total	5248	2948	337	675	738	1043	1355	1100	1278	1273	1292	1405	1823	1847	1578
	(100%)	(56%)	(6%)	(13%)	(14%)	(20%)	(26%)	(21%)	(24%)	(24%)	(24%)	(27%)	(35%)	(35%)	(30%)

### Potential prevention impact comparing health and capability across disease and healthy population groups

**Fig 1:**
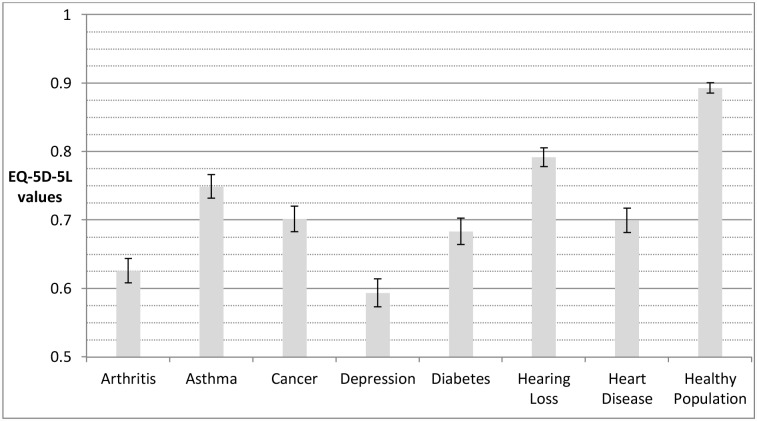
Disease groups and healthy population means for health (assessed by EQ-5D-5L). The error bars represent 95% confidence intervals around the mean. EQ-5D-5L values generated using cross-walk from EQ-5D-3L UK value set, based on time trade-off [31].

**Fig 2:**
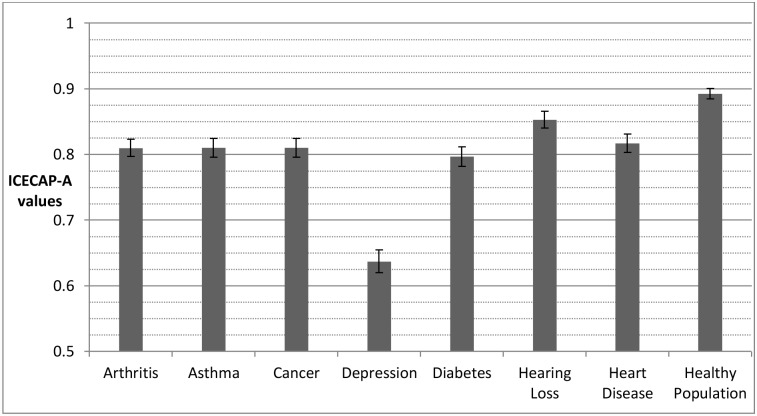
Disease groups and healthy population means for capability (assessed by ICECAP-A). The error bars represent 95% confidence intervals around the mean. ICECAP-A values generated from original UK population tariff, based on best-worst scaling [37].

Depression results in the lowest mean state whether individuals’ outcomes are measured using the EQ-5D-5L (Fig 1) or the ICECAP-A (Fig 2). For other conditions, however, the difference relative to healthy individuals varies depending on whether capability or health is measured. For example, the capability status for individuals with arthritis is close to the capability status for the healthy population. Yet, the health status of individuals with arthritis is substantially below the health status of the healthy population and indeed it is closer to the health status for individuals with depression.

Table 3 reports effect sizes for the mean outcome of disease groups relative to the healthy population. For both outcome measures, and across all diseases, those with diseases score significantly (p<0.05) worse than the healthy population. The absolute effect sizes for the impact of the conditions are greater for health in all cases than for capability, although the relative differences vary considerably. In relation to capability, the effect size for depression is much greater (1.22) than for all other conditions. Effect sizes for arthritis, asthma, cancer and diabetes and heart disease are similar (0.49 to 0.59) and the effect size in relation to hearing loss is lower (0.28).

**Table 3:** Effect sizes for disease groups compared to the healthy population for capability (assessed by ICECAP-A) and health (assessed by EQ-5D-5L).

	EFFECT SIZES		ORDERING OF CONDITIONS BY EFFECT SIZE	
	Capability	Health	Capability	Health
ARTHRITIS	0.55**	1.24***	3	2
ASTHMA	0.55**	0.82***	3	6
CANCER	0.54**	1.00***	5	3
DEPRESSION	1.22***	1.26***	1	1
DIABETES	0.59**	0.99***	2	4
HEARING LOSS	0.28*	0.68**	7	7
HEART DISEASE	0.49*	0.99***	6	4

*small effect size (0.2+);

**medium effect size (0.5+);

***large effect size (0.8+)

For health there is a different pattern. Although the effect size for depression (1.26) is also highest (and of a similar order to that of capability), the effect size for arthritis is only very slightly lower (1.24). Effect sizes for cancer, diabetes and heart problems are also high and very similar in size (0.99 to 1.00), with asthma and hearing loss both having lower effect sizes (0.82 to 0.68).

The major conclusion from Table 3, however is that there are notable differences in the pattern of effect sizes across health conditions when measured by health and capability.

### Potential treatment impact comparing health and capability for different severities of condition

Individuals were allocated to mild, moderate or severe categories of the relevant disease group according to the global scores developed from condition-specific measures (see S3 Appendix for country breakdown). Numbers in each category differed by disease group, with four conditions (asthma, cancer, diabetes and heart disease) having the highest concentrations of individuals in the mild category and three (arthritis, depression and hearing loss) having the highest concentrations of individuals in the moderate category. Across all conditions, the depression category had the highest, and heart disease the lowest, number of individuals categorised as severe (150 and 45 respectively).

**Fig 3:**
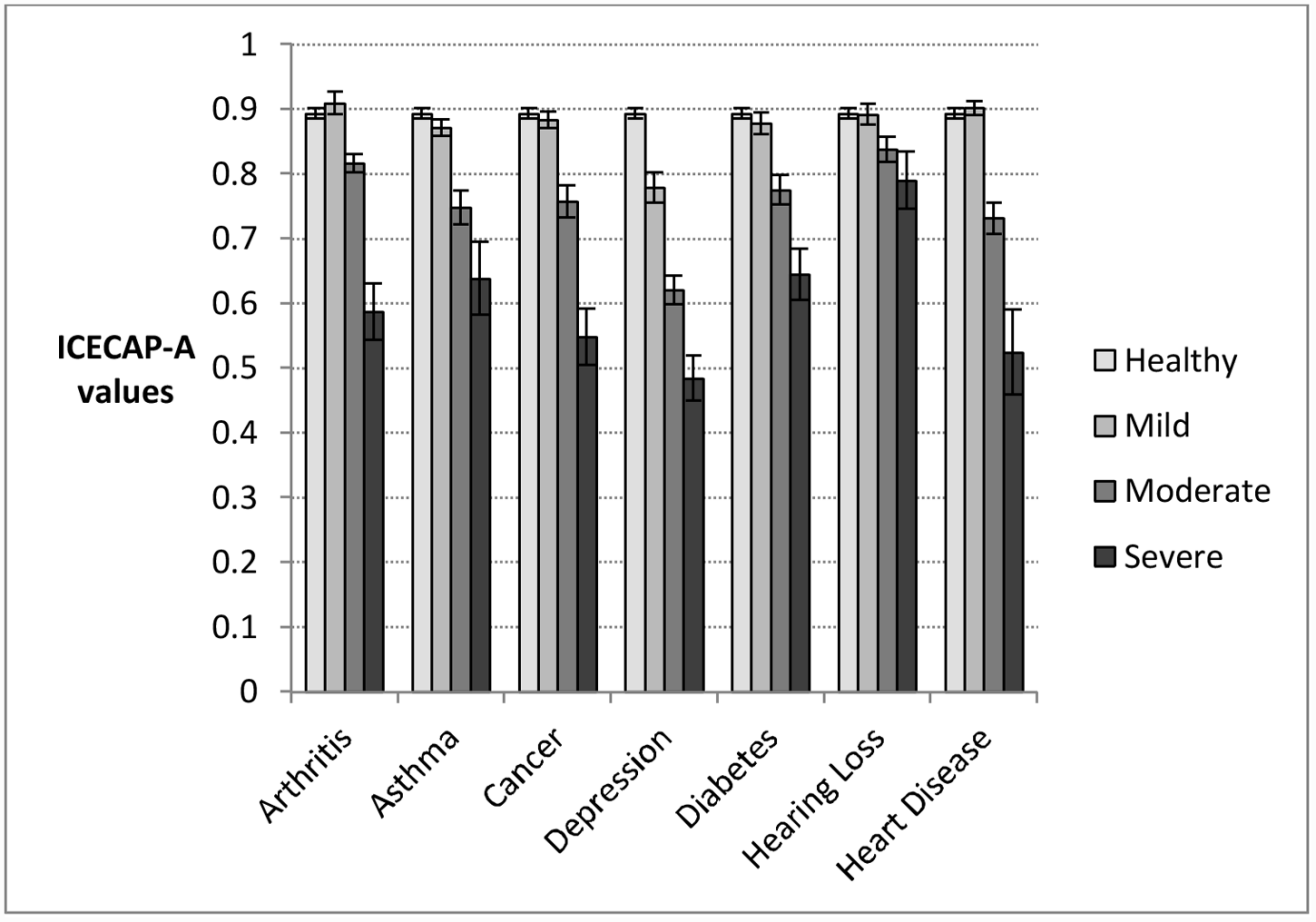
Disease group means and 95% confidence intervals for capability (assessed by ICECAP-A), by severity category according to global score derived from condition-specific measures. Population category sample sizes: Arthritis: mild, n = 132; moderate, n = 437; severe, n = 71; Asthma: mild, n = 344; moderate, n = 177; severe, n = 58; Cancer, mild: n = 342; moderate, n = 175; severe, n = 60; Depression: mild, n = 195; moderate, n = 272; severe, n = 150; Diabetes: mild, n = 277; middle, n = 253; severe, n = 111; Hearing Loss: mild, n = 232; moderate, n = 286; severe, n = 71; Heart disease: mild, n = 378; moderate, n = 217; severe, n = 45; Healthy population: n = 965.

**Fig 4:**
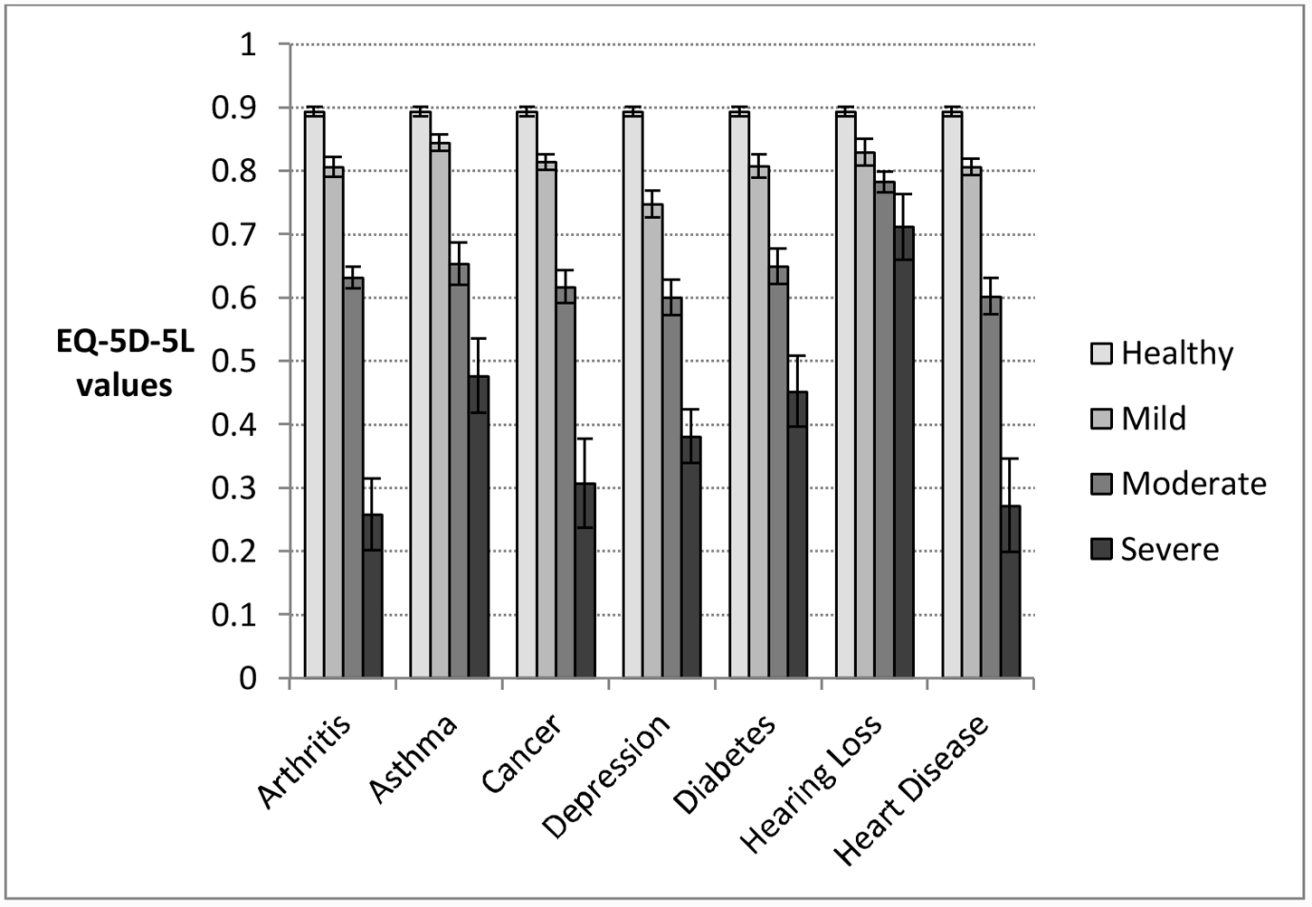
Disease group means and 95% confidence intervals for health (assessed by EQ-5D-5L), by severity category according to global score derived from condition-specific measures. Population category sample sizes: Arthritis: mild, n = 132; moderate, n = 437; severe, n = 71; Asthma: mild, n = 344; moderate, n = 177; severe, n = 58; Cancer, mild: n = 342; moderate, n = 175; severe, n = 60; Depression: mild, n = 195; moderate, n = 272; severe, n = 150; Diabetes: mild, n = 277; middle, n = 253; severe, n = 111; Hearing Loss: mild, n = 232; moderate, n = 286; severe, n = 71; Heart disease: mild, n = 378; moderate, n = 217; severe, n = 45; Healthy population: n = 965.

There was little difference in the capability score between individuals with mild arthritis, hearing loss, or heart disease and the healthy population (Fig 3). Depression is the only disease group where individuals in the mildly severe group have capability values that differ significantly from the healthy population (0.779). In contrast, individuals in all of the mild health categories have reduced health compared to the healthy population (Fig 4). While the value for the group with depression is also low (0.748), for other conditions the values range between 0.806 (arthritis) and 0.844 (asthma). The highest of these values is still 0.05 below the healthy population value.

There also appear to be differences between health and capability for those who have the most severe conditions. The lowest capability scores are obtained by those with severe depression (0.484), severe cancer (0.548) and severe arthritis (0.587). The lowest health scores are for those with severe arthritis (0.257), severe heart disease (0.272) and severe cancer (0.307).

These scores are, again, based on different scales. Apart from individuals with depression, mildly severe individuals and the healthy group fail to have a small effect size in terms of capability; for depression the effect size is large (Table 4). Between the severe and moderate categories, and the moderate and mild categories, effect sizes are all moderate or large, except for hearing loss. In contrast with capabilities all of the results from the health scale exceed the cut off score for a small effect size. The differences between the mild and healthy groups have small or moderate effect sizes for all conditions apart from depression, where the effect size is large. Between the severe and moderate categories, and the moderate and mild categories, effect sizes are all moderate or large, except for the case of hearing loss where they are again small.

**Table 4:** Effect size for differences in mean capability (ICECAP-A) and health status (EQ-5D-5L) between mild, moderate and severe impairment in seven diseases.

Effect Sizes	Arthritis	Asthma	Cancer	Depression	Diabetes	Hearing Loss	Heart Disease
**MILD VERSUS HEALTHY**							
Capability	-0.13	0.17	0.07	0.82***	0.12	0.01	-0.07
Health	0.72**	0.40*	0.63**	1.06***	0.64**	0.48*	0.68**
Sample size	1,097	1,309	1,307	1,160	1,242	1,197	1,343
**MODERATE VERSUS MILD**							
Capability	0.63**	0.79**	0.84***	0.81***	0.60**	0.36*	1.07***
Health	0.97***	1.01***	1.17***	0.68**	0.75**	0.30*	1.07***
Sample size	569	521	517	467	530	518	595
**SEVERE VERSUS MODERATE**							
Capability	1.31***	0.56**	1.19***	0.65**	0.65**	0.27*	1.01***
Health	1.62***	0.74**	1.26***	0.81***	0.73**	0.44*	1.30***
Sample size	508	235	235	422	364	357	265

Categories on a 1–0 mild severe scale for each condition-specific questionnaire where mild, > 0.7; moderate, 0.4–0.7; severe.<0.4. Effect sizes:

small (*) = 0.2+;

medium effect size (**) = 0.5+;

large effect size (***) = 0.8+.

Reported sample size is combination of two groups under consideration. Sensitivity analysis using US weights for EQ-5D-5L are reported in S4 Appendix.

## Discussion

The findings reported here show that major diseases are associated with relatively different impacts on health and capability. This indicates that a focus on capability outcomes, rather than with health status, would alter the relative importance of preventing and treating different conditions. In particular, two findings from this research are significant. The first is the relatively greater effect sizes on the capability scale for individuals with depression as compared with other disease groups. The second is the insignificant difference in effect sizes for capabilities between individuals with mild conditions and the healthy population for all of the disease groups apart from depression.

To date, empirical comparisons of the capability approach with respect to health are relatively limited to single disease contexts [20,51–56], reflecting the lack of large datasets across multiple conditions. This paper therefore provides the first evidence for the possibility that different inferences may be drawn about the relative value of treating and preventing different conditions across the health service when focusing on improving capability wellbeing rather than health status.

There are, however, a number of limitations. First, the MIC dataset is cross-sectional, and inferences about the impact of diseases are made by comparing disease groups and healthy members of the general population. This is an inevitable limitation of research until time series data becomes available. Second, the global scoring system used for categorising individuals produced a consistent method of grouping across disease areas, but is inescapably somewhat arbitrary as there is no recognised method of classification for all diseases. The need for cross disease group analyses highlights the importance of generic instruments such as EQ-5D-5L [29], and ICECAP-A [35]. It is important to acknowledge that the ICECAP-A is one interpretation of measuring capability, and other measures of capability have also been recently developed [57–64]. Finally both instruments were scored using UK values as only UK values exist for ICECAP-A. Values are available for EQ-5D-5L for the USA but not for Australia or Canada. The US values for EQ-5D-5L were employed in a separate analysis but resulted in almost identical findings (see S4 Appendix).

Despite these limitations, the present study indicates how interventions for different health conditions might be prioritised under a different evaluative paradigm and there are a number of implications of this. First, with effective interventions, preventing morbidity from an average case of depression would have a significantly larger impact on capability wellbeing than preventing morbidity in any of the other disease areas in this study. The relative priority given to effective treatments for depression would therefore rise under this paradigm. Second, the findings suggest that the impact on capability wellbeing of mild disease is generally very small. This suggests that, by contrast with evaluations based upon health there may be little benefit from interventions focused on preventing mild morbidity. The exception to this is depression, where even mild disease appears to have a clear impact on an individual’s capability. Prioritising on the basis of capability, therefore, suggests that greater priority would go to those with depression and those with severe or moderate illness than under a paradigm where the evaluative focus was on health status.

The results suggest a number of avenues for further research. First, more information is needed both from time series and intervention studies. Second, these findings relate only to morbidity and not mortality. It has been estimated that around half the burden of poor health in one developed country setting, Australia, arises from morbidity rather than premature mortality [23]. For some of the conditions explored here there is very high premature mortality, with around one third of the burden of premature mortality terms arising from cancer and a similar amount from heart disease [23]. A comprehensive system of prioritising must, of course, also take account of this. Third, alternative measures of both health and capability, and indeed health capability [8,65] could be used to assess the extent to which the results reported here are influenced by the particular choices of health status and capability instruments. The EQ-5D values are particularly sensitive to pain and physical problems [22]. Other instruments with a greater psycho-social content might result in different conclusions. Conversely a person’s perception of their capability in different areas of their life may be affected by their mood [66]. It would also be worth exploring further if depression affected capability similarly across different capability instruments. Depression’s large relative impact on capability may also influence individual’s capability with multiple morbidities, so this warrants further scrutiny in studies that collect data on more than one condition. Finally, a choice between prioritising on the basis of health or on the basis of capability wellbeing is ultimately a normative question. Societal views about such prioritisation methods should be sought.

To conclude, this study highlights the potential importance of the choice of outcome for the allocation of resources in the health sector. The suggestion from this work is that a shift from a focus on health to a broader focus on capability wellbeing could result in changes both to the disease areas that are given priority and the priority given to those with different levels of severity of the same disease.

## Supporting Information

S1 Appendix Calculation of Global Scores for Patient. (DOCX)

S2 Appendix Internal validation of global score categories. (DOCX)

S3 Appendix Further Country breakdown. (DOCX)

S4 Appendix Sensitivity analysis of health effect sizes. (DOCX)

## References

[1] Rumbold B, Alakeson V, Smith PC (2012) Rationing Health Care: Is it time to set out more clearly what is funded by the NHS? London: The Nuffield Trust.

[2] Arnold D, Girling A, Stevens A, Lilford R (2009) Comparison of direct and indirect methods of estimating health state utilities for resource allocation: review and empirical analysis. BMJ 339. 10.1136/bmj.b2688 PMC2714630 22128393

[3] Dolan P, Kahneman D (2008) Interpretations Of Utility And Their Implications For The Valuation Of Health*. The Economic Journal 118: 215–234.

[4] Nussbaum M (2011) Creating Capabilities: The Human Development Approach. Cambridge, MA: Belknap.

[5] Ruger JP (2010) Health And Social Justice. Oxford, UK: Oxford University Press.

[6] Sen A (1992) Inequality Reexamined Great Clarendon Street, Oxford: Oxford University Press.

[7] Sen A (1993) Capability and Well-Being In: Nussbaum MC, Sen A, editors. The Quality of Life. Great Clarendon Street, Oxford: Oxford University Press pp. 30–53.

[8] Venkatapuram S (2011) Health Justice. Cambridge, UK: Polity Press.

[9] Sen A (2006) Reason, Freedom and Well-being. Utilitas 18: 80–96.

[10] Sen A (2009) The Idea of Justice. London: Allen Lane.

[11] Ariana P, Naveed A (2009) Health In: Deneulin S, Shahani L, editors. An Introduction to the Human Development and Capability Approach. London: Earthscan pp. 228–245.

[12] Coast J, Kinghorn P, Mitchell P (2015) The Development of Capability Measures in Health Economics: Opportunities, Challenges and Progress. The Patient—Patient-Centered Outcomes Research 8: 119–126. 10.1007/s40271-014-0080-1 25074355

[13] Coast J, Smith R, Lorgelly P (2008) Should the capability approach be applied in Health Economics? Health Economics 17: 667–670. 10.1002/hec.1359 18457341

[14] Entwistle VA, Watt IS (2013) Treating Patients as Persons: A Capabilities Approach to Support Delivery of Person-Centered Care. The American Journal of Bioethics 13: 29–39. 10.1080/15265161.2013.802060 PMC3746461 23862598

[15] Hausman DM (2010) Valuing health: a new proposal. Health Economics 19: 280–296. 10.1002/hec.1474 19301348

[16] Smith R, Lorgelly P, Al-Janabi H, Venkatapuram S, Coast J (2012) The capability approach: an alternative evaluation paradigm for health economics? In: Jones AM, editor. The Elgar Companion to Health Economics. 2nd ed Cheltenham, UK: Edward Elgar Publishing Limited pp. 415–424.

[17] Wolff J, Edwards S, Richmond S, Orr S, Rees G (2012) Evaluating interventions in health: A reconcilatory approach. Bioethics 26: 455–463. 10.1111/j.1467-8519.2011.01888.x 21535065

[18] Davis J, Bryan S, McLeod R, Rogers J, Khan K, Liu-Ambrose T (2012) Exploration of the association between quality of life, assessed by the EQ-5D and ICECAP-O, and falls risk, cognitive function and daily function, in older adults with mobility impairments. BMC Geriatrics 12: 65 10.1186/1471-2318-12-65 23095570 PMC3534357

[19] Henderson C, Knapp M, Fernández J-L, Hirani SP, Cartwright M, Rixon L, et al (2013) Cost effectiveness of telehealth for patients with long term conditions (Whole Systems Demonstrator telehealth questionnaire study): nested economic evaluation in a pragmatic, cluster randomised controlled trial. BMJ 346. 10.1136/bmj.f1035 23520339

[20] Makai P, Looman W, Adang E, Melis R, Stolk E, Fabbricotti I (2015) Cost-effectiveness of integrated care in frail elderly using the ICECAP-O and EQ-5D: does choice of instrument matter? The European Journal of Health Economics 16: 437–450. 10.1007/s10198-014-0583-7 24760405

[21] Ratcliffe J, Lester LH, Couzner L, Crotty M (2013) An assessment of the relationship between informal caring and quality of life in older community-dwelling adults—more positives than negatives? Health & Social Care in the Community 21: 35–46. 22812476 10.1111/j.1365-2524.2012.01085.x

[22] Richardson J, Khan MA, Iezzi A, Maxwell A (2015) Comparing and Explaining Differences in the Magnitude, Content, and Sensitivity of Utilities Predicted by the EQ-5D, SF-6D, HUI 3, 15D, QWB, and AQoL-8D Multiattribute Utility Instruments. Medical Decision Making 35: 276–291. 10.1177/0272989X14543107 25159172

[23] Department of Human Services (2005) Victorian Burden of Disease Study Mortality and morbidity in 2001. Australia: Victorian Government Department of Human Services.

[24] Vos T, Barber RM, Bell B, Bertozzi-Villa A, Biryukov S, Bolliger I, et al (2015) Global, regional, and national incidence, prevalence, and years lived with disability for 301 acute and chronic diseases and injuries in 188 countries, 1990–2013: a systematic analysis for the Global Burden of Disease Study 2013. The Lancet 386: 743–800. 10.1016/S0140-6736(15)60692-4 PMC4561509 26063472

[25] The EuroQol Group (1990) EuroQol—a new facility for the measurement of health-related quality of life. Health Policy 16: 199–208. 10109801 10.1016/0168-8510(90)90421-9

[26] Brooks R (1996) EuroQol: the current state of play. Health Policy 37: 53–72. 10158943 10.1016/0168-8510(96)00822-6

[27] Wisløff T, Hagen G, Hamidi V, Movik E, Klemp M, Olsen J (2014) Estimating QALY Gains in Applied Studies: A Review of Cost-Utility Analyses Published in 2010. PharmacoEconomics 32: 367–375. 10.1007/s40273-014-0136-z 24477679 PMC3964297

[28] Devlin N, Krabbe PM (2013) The development of new research methods for the valuation of EQ-5D-5L. The European Journal of Health Economics 14: 1–3. 23900659 10.1007/s10198-013-0502-3 PMC3728454

[29] Herdman M, Gudex C, Lloyd A, Janssen MF, Kind P, Parkin D, et al (2011) Development and preliminary testing of the new five-level version of EQ-5D (EQ-5D-5L). Quality of Life Research 20: 1727–1736. 10.1007/s11136-011-9903-x 21479777 PMC3220807

[30] Dolan P (1997) Modeling Valuations for EuroQol Health States. Medical Care 35: 1095–1108. 9366889 10.1097/00005650-199711000-00002

[31] van Hout B, Janssen MF, Feng Y-S, Kohlmann T, Busschbach J, Golicki D, et al (2012) Interim Scoring for the EQ-5D-5L: Mapping the EQ-5D-5L to EQ-5D-3L Value Sets. Value in Health 15: 708–715. 10.1016/j.jval.2012.02.008 22867780

[32] NICE (2012) Assessing cost effectiveness The guidelines manual. London: National Institute for Health and Care Excellence pp. 107–122.

[33] Russell LB, Gold MR, Siegel JE, Daniels N, Weinstein MC (1996) The role of cost-effectiveness analysis in health and medicine. JAMA 276: 1172–1177. 8827972

[34] Janssen MF, Pickard AS, Golicki D, Gudex C, Niewada M, Scalone L, et al (2013) Measurement properties of the EQ-5D-5L compared to the EQ-5D-3L across eight patient groups: a multi-country study. Quality of Life Research 22: 1717–1727. 10.1007/s11136-012-0322-4 23184421 PMC3764313

[35] Al-Janabi H, Flynn T, Coast J (2012) Development of a self-report measure of capability wellbeing for adults: the ICECAP-A. Quality of Life Research 21: 167–176. 10.1007/s11136-011-9927-2 21598064 PMC3254872

[36] Cookson R (2005) QALYs and the capability approach. Health Economics 14: 817–829. 15693028 10.1002/hec.975

[37] Flynn TN, Huynh E, Peters TJ, Al-Janabi H, Clemens S, Moody A, et al (2015) Scoring the ICECAP-A Capability Instrument. Estimation of a UK General Population Tariff. Health Economics 24: 258–269. 10.1002/hec.3014 24254584 PMC4322472

[38] Dagsvik J (2013) Making Sen’s capability approach operational: a random scale framework. Theory and Decision 74: 75–105.

[39] Al-Janabi H, Peters T, Brazier J, Bryan S, Flynn T, Clemens S, et al (2013) An investigation of the construct validity of the ICECAP-A capability measure. Quality of Life Research 22: 1831–1840. 10.1007/s11136-012-0293-5 23086535 PMC3764327

[40] Guillemin F, Coste J, Pouchot J, Ghézail M, Bregeon C, Sany J (1997) The AIMS2-SF. A short form of the arthritis impact measurement scales 2. Arthritis & Rheumatism 40: 1267–1274. 9214427 10.1002/1529-0131(199707)40:7<1267::AID-ART11>3.0.CO;2-L

[41] Marks GB, Dunn SM, Woolcock AJ (1992) A scale for the measurement of quality of life in adults with asthma. Journal of Clinical Epidemiology 45: 461–472. 1588352 10.1016/0895-4356(92)90095-5

[42] Aaronson NK, Ahmedzai S, Bergman B, Bullinger M, Cull A, Duez NJ, et al (1993) The European Organization for Research and Treatment of Cancer QLQ-C30: A Quality-of-Life Instrument for Use in International Clinical Trials in Oncology. Journal of the National Cancer Institute 85: 365–376. 8433390 10.1093/jnci/85.5.365

[43] Lovibond SH, Lovibond PF (1995) Manual for the Depression Anxiety Stress Scales. Sydney, Australia: Psychology Foundation.

[44] Kessler RC, Barker PR, Colpe LJ, et al (2003) Screening for serious mental illness in the general population. Archives of General Psychiatry 60: 184–189. 12578436 10.1001/archpsyc.60.2.184

[45] Boyer JG, Earp JAL (1997) The Development of an Instrument for Assessing the Quality of Life of People with Diabetes: Diabetes-39. Medical Care 35: 440–453. 9140334 10.1097/00005650-199705000-00003

[46] Cox RM, Alexander GC (1995) The Abbreviated Profile of Hearing Aid Benefit. Ear and Hearing 16: 176–186. 7789669 10.1097/00003446-199504000-00005

[47] Höfer S, Lim L, Guyatt G, Oldridge N (2004) The MacNew Heart Disease health-related quality of life instrument: A summary. Health and Quality of Life Outcomes 2: 1–8. 14713315 10.1186/1477-7525-2-3 PMC341459

[48] Cohen J (1988) Statistical Power Analysis for the Behavioural Sciences. 2nd ed New York: Psychology Press.

[49] Walters S, Brazier J (2005) Comparison of the minimally important difference for two health state utility measures: EQ-5D and SF-6D. Quality of Life Research 14: 1523–1532. 16110932 10.1007/s11136-004-7713-0

[50] ten Klooster PM, Veehof MM, Taal E, van Riel PLCM, van de Laar MAFJ (2008) Confirmatory factor analysis of the Arthritis Impact Measurement Scales 2 short form in patients with rheumatoid arthritis. Arthritis Care & Research 59: 692–698. 18438904 10.1002/art.23569

[51] Comans T, Peel N, Gray L, Scuffham P (2013) Quality of life of older frail persons receiving a post-discharge program. Health and Quality of Life Outcomes 11: 1–7. 23587460 10.1186/1477-7525-11-58 PMC3637078

[52] Couzner L, Crotty M, Norman R, Ratcliffe J (2013) A Comparison of the EQ-5D-3L and ICECAP-O in an Older Post-Acute Patient Population Relative to the General Population. Applied Health Economics and Health Policy 11: 415–425. 10.1007/s40258-013-0039-8 23807538

[53] Davis J, Liu-Ambrose T, Richardson C, Bryan S (2013) A comparison of the ICECAP-O with EQ-5D in a falls prevention clinical setting: are they complements or substitutes? Quality of Life Research 22: 969–977. 10.1007/s11136-012-0225-4 22723152 PMC3672090

[54] Keeley T, Al-Janabi H, Nicholls E, Foster NE, Jowett S, Coast J (2015) A longitudinal assessment of the responsiveness of the ICECAP-A in a randomised controlled trial of a knee pain intervention. Quality of Life Research: 1–13. 10.1007/s11136-015-0980-0 PMC4564441 25894061

[55] Parsons N, Griffin XL, Achten J, Costa ML (2014) Outcome assessment after hip fracture: is EQ-5D the answer? Bone and Joint Research 3: 69–75. 10.1302/2046-3758.33.2000250 24648420 PMC3963508

[56] van Leeuwen KM, Bosmans JE, Jansen APD, Hoogendijk EO, van Tulder MW, van der Horst HE, et al (2015) Comparing Measurement Properties of the EQ-5D-3L, ICECAP-O, and ASCOT in Frail Older Adults. Value in Health 18: 35–43. 10.1016/j.jval.2014.09.006 25595232

[57] Anand P, Hunter G, Carter I, Dowding K, Guala F, Van Hees M (2009) The Development of Capability Indicators. Journal of Human Development and Capabilities 10: 127.

[58] Coast J, Flynn TN, Natarajan L, Sproston K, Lewis J, Louviere JJ, et al (2008) Valuing the ICECAP capability index for older people. Social Science & Medicine 67: 874–882. 18572295 10.1016/j.socscimed.2008.05.015

[59] Greco G, Skordis-Worrall J, Mkandawire B, Mills A (2015) What is a good life? Selecting capabilities to assess women's quality of life in rural Malawi. Social Science & Medicine 130: 69–78. 25687242 10.1016/j.socscimed.2015.01.042

[60] Kinghorn P, Robinson A, Smith R (2015) Developing a Capability-Based Questionnaire for Assessing Well-Being in Patients with Chronic Pain. Social Indicators Research 120: 897–916.

[61] Lorgelly PK, Lorimer K, Fenwick E, Briggs AH, Anand P (2015) Operationalising the Capability Approach as an Outcome Measure in Public Health: the development of the OCAP-18. Social Science & Medicine 142: 68–81. 26291444 10.1016/j.socscimed.2015.08.002

[62] Netten A, Burge P, Malley J, Potoglou D, Towers A-M, Brazier J, et al (2012) Outcomes of social care for adults: developing a preference-weighted measure. Health Technology Assessment 16: 1–166. 10.3310/hta16160 22459668

[63] Simon J, Anand P, Gray A, Rugkåsa J, Yeeles K, Burns T (2013) Operationalising the capability approach for outcome measurement in mental health research. Social Science & Medicine 98: 187–196. 24331898 10.1016/j.socscimed.2013.09.019

[64] Sutton EJ, Coast J (2014) Development of a supportive care measure for economic evaluation of end-of-life care using qualitative methods. Palliative Medicine 28: 151–157. 10.1177/0269216313489368 23698452

[65] Ruger JP (2010) Health Capability: Conceptualization and Operationalization. American Journal of Public Health 100: 41–49. 10.2105/AJPH.2008.143651 19965570 PMC2791246

[66] Al-Janabi H, Keeley T, Mitchell P, Coast J (2013) Can capabilities be self-reported? A think aloud study. Social Science & Medicine 87: 116–122. 23631786 10.1016/j.socscimed.2013.03.035 PMC3664929

