# Supplementary material for: The Relative Impacts of Disease on Health Status and Capability Wellbeing: A Multi-Country Study
Source: PLoS One. 2015 Dec 2;10(12):e0143590. doi: 10.1371/journal.pone.0143590 (PMC4667875; doi:10.1371/journal.pone.0143590)
Supplement: S1 Appendix — (DOCX) [file pone.0143590.s001.docx]

## S1 Appendix. Calculation of Global Scores for Patient Groups

#### Arthritis

The Arthritis Impact Measurement Scale 2 – Short Form (AIMS2-SF)[[35](#_ENREF_35)] global score was calculated by adding the five mean subscales together. In cases where an individual did not work, the work subscale was omitted and the mean of the four remaining subscales was used to calculate the global score.

#### Asthma

The Asthma Quality of Life Questionnaire (AQLQ-Sydney) global score was calculated as the mean of the total twenty items on the questionnaire [[36](#_ENREF_36)].

#### Cancer

The team behind the European Organisation for Research and Treatment of Cancer Quality of Life Questionnaire Cancer 30 (EORTC QLQ-C30) do not recommend global mean scores to be calculated from items or subscales[[37](#_ENREF_37)]. However, for the purposes of this study this was necessary. Both a combination of the 28 items into a mean score and a mean of the combined subscale scores were tested. The mean of the combined subscale scores was selected on the grounds that it led to a closer equal distribution across the three severity levels.

#### Depression

The Depression Anxiety and Stress Scale (DASS21)[[38](#_ENREF_38)] was selected from the two available measures due to its broader focus on anxiety and stress. The global score is the mean of all DASS21 items or the mean of the three subscales (equivalent).

#### Diabetes

The Diabetes-39 questionnaire[[40](#_ENREF_40)] global score was calculated by the mean of all 39 items, as was done in a previous study with the questionnaire [[47](#_ENREF_47)].

#### Hearing Loss

The Abbreviated profile of hearing aid benefit (APHAB)[[41](#_ENREF_41)] mean scores for the two parts of the questionnaire are calculated by the mean of three of the four subscales [[48](#_ENREF_48)]. The two parts of the APHAB were weighted so that:

- Those with less than 6 weeks or no hearing aid experience or used or used a hearing aid for less than one hour per day: score of APHAB without hearing aid only
- Those with more than 6 weeks of hearing aid experience & used hearing aid for 1 to 4 hours per day:2/3 weight of APHAB without hearing aid; 1/3 APHAB with hearing aid
- Those with more than 6 weeks of hearing aid experience & use hearing aid for 4 to 8 hours per day: 1/3 weight of APHAB without hearing aid; 2/3 APHAB with hearing aid
- Those with more than 6 weeks of hearing aid experience & use hearing aid for 8 to 16 hours per day: score of APHAB with hearing aid only

To compute the relevant subscales, each scale needed at least two of the six questions to be completed. For those who had a missing scale in (ii) or (iii), if that missing scale was in the less weighted side, the mean from the same scale on the other part of the questionnaire was used instead. When this was reversed, or when scales were missing from (i) and (iv), observations were excluded from further analysis.

#### Heart Disease

The global score of the MacNew was calculated as the mean of all 27 items included in that questionnaire [[42](#_ENREF_42)].
