# Supplementary material for: The Relative Impacts of Disease on Health Status and Capability Wellbeing: A Multi-Country Study
Source: PLoS One. 2015 Dec 2;10(12):e0143590. doi: 10.1371/journal.pone.0143590 (PMC4667875; doi:10.1371/journal.pone.0143590)
Supplement: S2 Appendix — (DOCX) [file pone.0143590.s002.docx]

## S2 Appendix. Internal validation of global score categories

### Introduction

To compare the relative impacts across different conditions, generic cutoffs of severity (i.e. mild, moderate and severe) are required, as not all of the condition specific questionnaires have pre-defined clinical cutoffs. The arthritis condition-specific questionnaire collected in this study, AIMS2-SF (see Table 1), has previously been used to compare condition severity across different studies. On a 0-1 scale where 0 is worst and 1 is best, severe was considered anything below 0.4, moderate was scores between 0.4 and 0.7 and mild was above 0.7 to 1 [[45](#_ENREF_45)]. In this additional analysis, the aim is to assess the validity of adopting this approach to setting global cutoffs, by comparing global cutoffs with clinically meaningful cutoffs for two depression condition-specific questionnaires.

### Methods

### Instruments

#### DASS21

The DASS21 consists of three categories of depression anxiety and stress, with seven questions for each category. Each question has four levels (does not apply to me at all – applies to me very much/most of the time) on a 0-3 scale (best-worst). Each question response is multiplied by two to give a DASS score which corresponds with the original 42 item DASS questionnaire, where a global score of 126 is the worst score possible on the questionnaire. As can be seen in S2 Table 1, five clinical cutoffs have been assigned to DASS-21 three categories [[38](#_ENREF_38)].

**S2 Table 1. DASS21 clinical category cutoffs**

| Severity | Depression | Anxiety | Stress | Clinical* |
| --- | --- | --- | --- | --- |
| Normal | 0-9 | 0-7 | 0-14 | 0-32 |
| Mild | 10-13 | 8-9 | 15-18 | 33-42 |
| Moderate | 14-20 | 10-14 | 19-25 | 43-61 |
| Severe | 21-27 | 15-19 | 26-33 | 62-81 |
| Very Severe | 28+ | 20+ | 34+ | 82+ |

## * We inferred clinical scores from the three categories on DASS-21

#### Kessler (K10)

The K10 consists of ten questions aimed to capture mental health disorders in the general population. Each question has five levels (None of the time-all of the time) on a 1-5 scale (best-worst). The 10 questions summated result in a score between 10-50. In S2 Table 2, four clinical cutoffs are associated with the scores on the K10 [[39](#_ENREF_39)].

## S2 Table 2. K10 clinical category cutoffs

| **Clinical score** | **Severity** |
| --- | --- |
| 10-19 | Likely to be well |
| 20-24 | Likely to have a mild depression and/or anxiety disorder |
| 25-29 | Likely to have a moderate depression and/or anxiety disorder |
| 30-50 | Likely to have a severe depression and/or anxiety disorder |

### Validating Global Cutoffs

To validate a global scoring approach of classifying patients into condition severity, the ability of the global cutoffs to classify severity between questionnaires is examined. Additionally the cross over from the already established clinical cutoffs for DASS21 and K10 is also assessed. It is hypothesised here that the use of the global cutoffs should be able to classify patients into severity groups similarly to the ability of the depression questionnaires to classify across the two clinical cutoffs for each questionnaire (e.g. is someone with mild depression on DASS-21 also classified as having mild depression on K10).

To compare the global cutoffs with the clinical cutoffs described above, predicting expected relationships were necessary prior to analysis as to the expected crossover between the different approaches. S2 Table 3 summarises the expected relationships between the two clinical cutoffs and the two global cutoffs for the questionnaires.

**S2 Table 3. Expected relationships between clinical and global cutoffs**

| DASS-21 CLINICAL | DASS-21 GLOBAL | K10 CLINICAL | K10 GLOBAL |
| --- | --- | --- | --- |
| NORMAL | MILD | WELL | MILD |
| MILD | MILD | MILD | MILD |
| MODERATE | MODERATE | MODERATE | MODERATE |
| SEVERE | SEVERE | SEVERE | SEVERE |
| VERY SEVERE | SEVERE | SEVERE | SEVERE |

DASS-21 CLINICAL, DASS-21 clinical cutoffs; DASS-21 GLOBAL, DASS-21 global cutoffs; K10 CLINICAL, K10 clincal cutoffs; K10 GLOBAL, K10 global cutoffs.

## Results

617 patients with depression completed the two condition-specific questionnaires in this study. The socio-demographic information of this population can be found in Table 2. The crossover from clinical cutoffs for K10 compared with clinical cutoffs DASS-21 is presented in S2 Table 4. All predictions as expected are in italics. 298 (48.30%) were predicted in the categories, with 181 (29.34%) one category away from what was expected a priori.

**S2 Table 4. DASS-21 and K10 clinical cutoff comparison**

| K10 CLINICAL |  | DASS-21 CLINICAL | | |  |  |
| --- | --- | --- | --- | --- | --- | --- |
|  | normal | mild | moderate | severe | very severe | total |
| well | *39* | 2 | 2 | 0 | 1 | 44 |
| mild | 43 | *12* | 12 | 1 | 0 | 68 |
| moderate | 40 | 20 | *19* | 8 | 1 | 88 |
| severe | 36 | 57 | 96 | *112* | *116* | 417 |
| total | 158 | 91 | 129 | 121 | 118 | 617 |

Expected relationships between clinical cutoffs marked in italics

In S2 Table 5, DASS-21 categorisation is compared between the clinical cutoffs and the global categories. Overall, 474 (76.82%) were accurately allocated to their expected categories.

**S2 Table 5. DASS-21 clinical and global cutoffs compared**

| DASS-21 GLOBAL |  | DASS-21 CLINICAL | | |  |  |
| --- | --- | --- | --- | --- | --- | --- |
|  | normal | mild | moderate | severe | very severe | total |
| mild | *158* | *37* | 0 | 0 | 0 | 195 |
| moderate | 0 | 54 | *129* | 89 | 0 | 272 |
| severe | 0 | 0 | 0 | *32* | *118* | 150 |
| total | 158 | 91 | 129 | 121 | 118 | 617 |

Expected relationships between cutoffs marked in italics

In S2 Table 6, K10 categorisation is compared between the clinical cutoffs and the global categories. The accuracy of expected relationships is reduced slightly from the same comparison with DASS-21 cutoffs (S2 Table 5), with 458 (74.23%) predicted relationships as expected.

**S2 Table 6. K10 clinical and global cutoffs compared**

| K10 GLOBAL | K10 CLINICAL | | | | |
| --- | --- | --- | --- | --- | --- |
|  | well | mild | moderate | severe | total |
| mild | *44* | *30* | 0 | 0 | 74 |
| moderate | 0 | 38 | *88* | 121 | 247 |
| severe | 0 | 0 | 0 | *296* | 296 |
| total | 44 | 68 | 88 | 417 | 617 |

Expected relationships between cutoffs marked in italics

In S2 Table 7, DASS-21 clincial cutoffs are compared with K10 global cutoffs. 321 (52.03%) of the patients are classed within the expected categories, an improvement on the clinical only expected categorization (see S2 Table 4).

**S2 Table 7. DASS-21 clinical and K10 global cutoffs compared**

| K10 GLOBAL |  | DASS-21 CLINICAL | | |  |  |
| --- | --- | --- | --- | --- | --- | --- |
|  | normal | mild | moderate | severe | very severe | total |
| mild | *57* | *10* | 6 | 0 | 1 | 74 |
| moderate | 90 | 45 | *64* | 33 | 15 | 247 |
| severe | 11 | 36 | 59 | *88* | *102* | 296 |
| total | 158 | 91 | 129 | 121 | 118 | 617 |

Expected relationships between cutoffs marked in italics

In S2 Table 8, DASS-21 global cutoffs are compared with K10 clinical cutoffs. 276 (44.73%) are in the expected categorisation, which is less than seen when comparing clinical cutoffs (see S2 Table 4). However, only 65 (10.53%) patients were more than once category away using the DASS-21 arbitrary cutoffs.

**S2 Table 8. DASS-21 global and K10 clinical categories compared**

| DASS-21 GLOBAL | K10 CLINICAL | | | | |
| --- | --- | --- | --- | --- | --- |
|  | well | mild | moderate | severe | total |
| mild | *39* | *49* | 47 | 60 | 195 |
| moderate | 4 | 19 | *40* | 209 | 272 |
| severe | 1 | 0 | 1 | *148* | 150 |
| total | 44 | 68 | 88 | 417 | 617 |

Expected relationships between cutoffs marked in italics

Finally, both global cutoffs are compared in S2 Table 9. Both of the arbitrary cutoffs produced expected classifications for both questionnaires for 302 (48.95%) of the patients in this sample, slightly better than the actual clinical cutoffs (S2 Table 4).

**S2 Table 9. DASS-21 and K10 global categories compared**

| K10 GLOBAL | DASS-21 GLOBAL | | | |
| --- | --- | --- | --- | --- |
|  | mild | moderate | severe | total |
| mild | *62* | 110 | 23 | 195 |
| moderate | 11 | *114* | 147 | 272 |
| severe | 1 | 23 | *126* | 150 |
| total | 74 | 247 | 296 | 617 |

Expected relationships between cutoffs marked in italics

## Conclusion

From the validation analysis undertaken, it is possible to say that the global cutoffs are classifying patients to a reasonable level, as the majority of global cutoffs produced better than expected predictions than when using the two clinical cutoffs for both questionnaires considered here. It is a limitation to extrapolate this validation to the rest of the dataset and patient populations. Nonetheless, it is a form of face validation of using the global classification of patient severity in the primary analysis.
