# Supplementary material for: The Relative Impacts of Disease on Health Status and Capability Wellbeing: A Multi-Country Study
Source: PLoS One. 2015 Dec 2;10(12):e0143590. doi: 10.1371/journal.pone.0143590 (PMC4667875; doi:10.1371/journal.pone.0143590)
Supplement: S3 Appendix — (DOCX) [file pone.0143590.s003.docx]

## S3 Appendix. Further Country breakdown

**S3 Table 1. Healthy sample EQ-5D-5L and ICECAP-A mean score per country**

|  | Australia | Canada | UK | US |
| --- | --- | --- | --- | --- |
| Sample size | 200 | 269 | 234 | 262 |
| EQ-5D-5L mean | 0.901 (0.105) | 0.895 (0.134) | 0.883 (0.117) | 0.894 (0.120) |
| ICECAP-A mean | 0.901 (0.113) | 0.890 (0.136) | 0.877 (0.135) | 0.903 (0.115) |

**Standard deviation in parentheses**

**S3 Table 2. Breakdown of patient severity categories across countries**

|  | Australia | Canada | UK | US |
| --- | --- | --- | --- | --- |
| ARTHRITIS |  |  |  |  |
| Mild | 46 (35%) | 13 (10%) | 41 (31%) | 32 (24%) |
| Moderate | 101 (23%) | 114 (26%) | 92 (21%) | 130 (30%) |
| Severe | 16 (23%) | 12 (17%) | 26 (37%) | 17 (24%) |
| Total | 163 (25%) | 129 (22%) | 159 (25%) | 179 (28%) |
| ASTHMA |  |  |  |  |
| Mild | 96 (28%) | 76 (22%) | 99 (29%) | 73 (21%) |
| Moderate | 37 (21%) | 48 (27%) | 41 (23%) | 51 (29%) |
| Severe | 8 (14%) | 14 (24%) | 10 (17%) | 26 (45%) |
| Total | 141 (24%) | 138 (24%) | 150 (26%) | 150 (26%) |
| CANCER |  |  |  |  |
| Mild | 87 (25%) | 89 (26%) | 72 (21%) | 94 (27%) |
| Moderate | 50 (29%) | 41 (23%) | 45 (26%) | 39 (22%) |
| Severe | 17 (28%) | 8 (13%) | 20 (33%) | 15 (25%) |
| Total | 154 (27%) | 138 (24%) | 137 (24%) | 148 (26%) |
| DEPRESSION |  |  |  |  |
| Mild | 49 (25%) | 43 (22%) | 46 (24%) | 57 (29%) |
| Moderate | 61 (22%) | 63 (23%) | 70 (26%) | 78 (29%) |
| Severe | 36 (24%) | 39 (26%) | 42 (28%) | 33 (22%) |
| Total | 146 (24%) | 145 (24%) | 158 (26%) | 168 (27%) |
| DIABETES |  |  |  |  |
| Mild | 68 (25%) | 60 (22%) | 85 (31%) | 64 (23%) |
| Moderate | 75 (30%) | 62 (25%) | 54 (21%) | 62 (25%) |
| Severe | 25 (23%) | 22 (20%) | 22 (20%) | 42 (38%) |
| Total | 168 (26%) | 144 (22%) | 161 (25%) | 168 (26%) |
| HEARING LOSS |  |  |  |  |
| Mild | 70 (30%) | 62 (27%) | 44 (19%) | 56 (24%) |
| Moderate | 73 (26%) | 71 (25%) | 59 (21%) | 83 (29%) |
| Severe | 14 (20%) | 13 (18%) | 23 (32%) | 21 (30%) |
| Total | 157 (27%) | 146 (25%) | 126 (21%) | 160 (27%) |
| HEART DISEASE |  |  |  |  |
| Mild | 98 (26%) | 95 (25%) | 90 (24%) | 95 (25%) |
| Moderate | 46 (21%) | 51 (24%) | 59 (27%) | 61 (28%) |
| Severe | 5 (11%) | 8 (18%) | 18 (40%) | 14 (31%) |
| Total | 149 (23%) | 154 (24%) | 167 (26%) | 170 (27%) |
