# Supplementary material for: The Relative Impacts of Disease on Health Status and Capability Wellbeing: A Multi-Country Study
Source: PLoS One. 2015 Dec 2;10(12):e0143590. doi: 10.1371/journal.pone.0143590 (PMC4667875; doi:10.1371/journal.pone.0143590)
Supplement: S4 Appendix — (DOCX) [file pone.0143590.s004.docx]

## S4 Appendix. Sensitivity analysis of health effect sizes

To test the reliability of the effect sizes results, Unites States values were used for the EQ-5D-5L health states. As only one valuation dataset exists for ICECAP-A, a sensitivity analysis could not be undertaken on these effect sizes.

In S4 Table 1, the results presented in table 3 on effect sizes between patient groups and the healthy public are supplemented by the effect sizes for the U.S. EQ-5D-5L population values. Changing to US EQ-5D-5L values from UK values results in no change to health effect sizes or order of patient group by effect size.

**S4 Table 1. Sensitivity analysis of effect sizes for patient groups compared to the healthy population**

|  | **EFFECT SIZES** | | | **ORDERING OF CONDITIONS BY**  **EFFECT SIZE** | | |
| --- | --- | --- | --- | --- | --- | --- |
|  | Capability | Health (UK) | Health (US) | Capability | Health (UK) | Health (US) |
| ARTHRITIS | 0.55** | 1.24*** | 1.22*** | 3 | 2 | 2 |
| ASTHMA | 0.55** | 0.82*** | 0.81*** | 3 | 6 | 6 |
| CANCER | 0.54** | 1.00*** | 0.99*** | 5 | 3 | 3 |
| DEPRESSION | 1.22*** | 1.26*** | 1.28*** | 1 | 1 | 1 |
| DIABETES | 0.59** | 0.99*** | 0.98*** | 2 | 4 | 4 |
| HEARING LOSS | 0.28* | 0.68** | 0.66** | 7 | 7 | 7 |
| HEART DISEASE | 0.49* | 0.99*** | 0.98*** | 6 | 4 | 4 |

*small effect size (0.2+); **medium effect size (0.5+); ***large effect size (0.8+)

In S4 Table 2, the results presented in table 4 on effect sizes between condition severity is supplemented with the effect sizes using US EQ-5D-5L values. As can be seen, all health values produce the same effect sizes irrespective of the values used for the EQ-5D-5L health states.

**S4 Table 2. Sensitivity analysis of condition severity effect size for ICECAP-A (UK) and EQ-5D-5L (UK & US values)**

| **Effect Sizes** | **Arthritis** | **Asthma** | **Cancer** | **Depression** | **Diabetes** | **Hearing Loss** | **Heart Disease** |
| --- | --- | --- | --- | --- | --- | --- | --- |
| **MILD VERSUS HEALTHY** | | |  | |  | | |
| Capability | -0.13 | 0.17 | 0.07 | 0.82*** | 0.12 | 0.01 | -0.07 |
| Health (UK) | 0.72** | 0.40* | 0.63** | 1.06*** | 0.64** | 0.48* | 0.68** |
| Health (US) | 0.69** | 0.38* | 0.61** | 1.05*** | 0.61** | 0.45* | 0.65** |
| **MODERATE VERSUS MILD** | | |  | |  | | |
| Capability | 0.63** | 0.79** | 0.84*** | 0.81*** | 0.60** | 0.36* | 1.07*** |
| Health (UK) | 0.97*** | 1.01*** | 1.17*** | 0.68** | 0.75** | 0.30* | 1.07*** |
| Health (US) | 0.97*** | 1.01*** | 1.17*** | 0.69** | 0.75** | 0.32* | 1.08*** |
| **SEVERE VERSUS MODERATE** | | |  | |  | | |
| Capability | 1.31*** | 0.56** | 1.09*** | 0.65** | 0.65** | 0.27* | 1.01*** |
| Health (UK) | 1.62*** | 0.74** | 1.26*** | 0.81*** | 0.73** | 0.44* | 1.30*** |
| Health (US) | 1.61*** | 0.77** | 1.26*** | 0.80*** | 0.74** | 0.40* | 1.32*** |
